# Path loss dataset for modeling radio wave propagation in smart campus environment

**DOI:** 10.1016/j.dib.2018.02.026

**Published:** 2018-02-16

**Authors:** Segun I. Popoola, Aderemi A. Atayero, Oghenekaro D. Arausi, Victor O. Matthews

**Affiliations:** Department of Electrical and Information Engineering, Covenant University, Ota, Nigeria

**Keywords:** Path loss, Radio propagation, Wireless communications, GSM networks, Smart campus

## Abstract

Path loss models are often used by radio network engineers to predict signal coverage, optimize limited network resources, and perform interference feasibility studies. However, the propagation mechanisms of electromagnetic waves depend on the physical characteristics of the wireless channel. Therefore, efficient radio network planning and optimization requires detailed information about the specific propagation environment. In this data article, the path loss data and the corresponding information that are needed for modeling radio wave propagation in smart campus environment are presented and analyzed. Extensive drive test measurements are performed along three different routes (X, Y, and Z) within Covenant University, Ota, Ogun State, Nigeria (Latitude 6°40′30.3″N, Longitude 3°09′46.3″E) to record path loss data as the mobile receiver moves away from each of the three 1800 MHz base station transmitters involved. Also, the longitude, latitude, elevation, altitude, clutter height, and the distance information, which describes the smart campus environment, are obtained from Digital Terrain Map (DTM) in ATOLL radio network planning tool. Results of the first-order descriptive statistics and the frequency distributions of all the seven parameters are presented in tables and graphs respectively. In addition, correlation analyses are performed to understand the relationships between the network parameters and the terrain information. For ease of reuse, the comprehensive data are prepared in Microsoft Excel spreadsheet and attached to this data article. In essence, the availability of these data will facilitate the development of path loss models for efficient radio network planning and optimization in smart campus environment.

**Specifications Table**

**Table:** 

Subject area	*Engineering*
More specific subject area	*Telecommunication Engineering*
Type of data	*Tables, graphs, figures, and spreadsheet file*
How data was acquired	*Measurement campaigns were carried out to obtain path loss data between GSM mobile station and three 1800* *MHz base station transmitters along three different routes within Covenant University, Ota, Ogun State, Nigeria (Latitude 6°40′30.3*″*N, Longitude 3°09′46.3*″*E). The data collection was performed using drive test approach.*
Data format	*Raw, analyzed*
Experimental factors	*Radio signal measurement and data collection processes were limited to the coverage areas of the directional transmitter antennas*
Experimental features	*Results of the first-order descriptive statistics and the frequency distributions of the network and terrain parameters are presented in tables and graphs respectively. In addition, correlation analyses are performed to understand the relationships between the network parameters and the terrain information*
Data source location	*Extensive drive test measurements are carried out along three different routes (X, Y, and Z) within Covenant University, Ota, Ogun State, Nigeria (Latitude 6°40′30.3*″*N, Longitude 3°09′46.3*″*E)*
Data accessibility	*The dataset on path loss and terrain information along the three survey routes are attached to this data article*

**Value of the data**

• Availability of the data in this data article will facilitate the development of path loss models for efficient radio network planning and optimization in smart campus environment [1], [2], [3], [4], [5], [6].
• Path loss data and terrain information provided in this article will aid comparative analysis and evaluation of existing and new empirical models [7], [8], [9], [10].
• In order to accurately account for the peculiarity of smart campus environment, existing path loss models may be tuned or re-calibrated using the data obtained from real scenarios [11], [12], [13].
• Achieving accurate path loss prediction within smart campus context will guarantee better Quality of Service (QoS) for smart applications [14], [15].
• The results of the correlation analyses will give better understanding about the relationships between the network parameters and the terrain information [16].
• The local content of the data may open doors of new research collaborations toward the development of a robust regional path loss model for wider coverage.

## 1 Data

In the present Information Age, high proliferation of smart devices that have in-built sensors and capabilities for Wireless Fidelity (Wi-Fi) and cellular wireless connectivity is fast changing the way things are done in university communities [11], [17]. A larger percentage of the activities that take place in university campuses are now extensively driven by Information and Communication Technologies (ICTs). Wireless communications provide the network infrastructures for seamless operations of smart applications within a smart campus environment [16]. Therefore, to guarantee good Quality of Service for smart applications within smart campus context, an efficient radio network planning and optimization procedures must be ensured [18]. Signal path loss models are used to predict the mean received signal strength of radio wave at specified distance of separation between the transmitting antenna and the receiving antenna [19], [20]. However, the propagation mechanisms of electromagnetic waves depend on the physical characteristics of the wireless channel. In order to accurately account for the peculiarity of smart campus environment, existing path loss models may be tuned or re-calibrated using the data obtained from real scenarios.

**Fig. 1:**
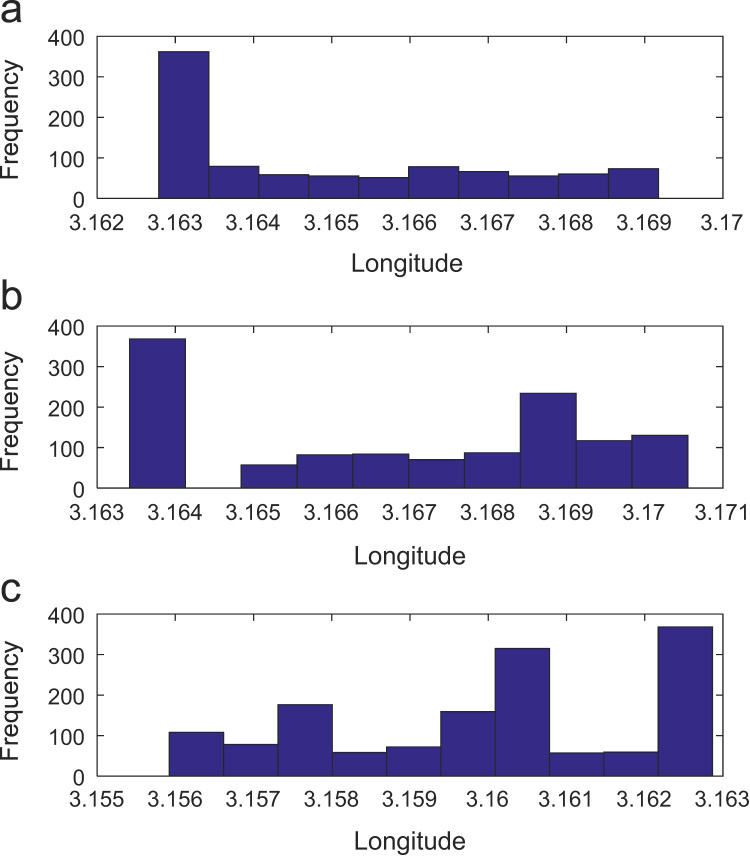
Frequency distribution of longitude data along Survey Route (a) X (b) Y and (c) Z.

**Fig. 2:**
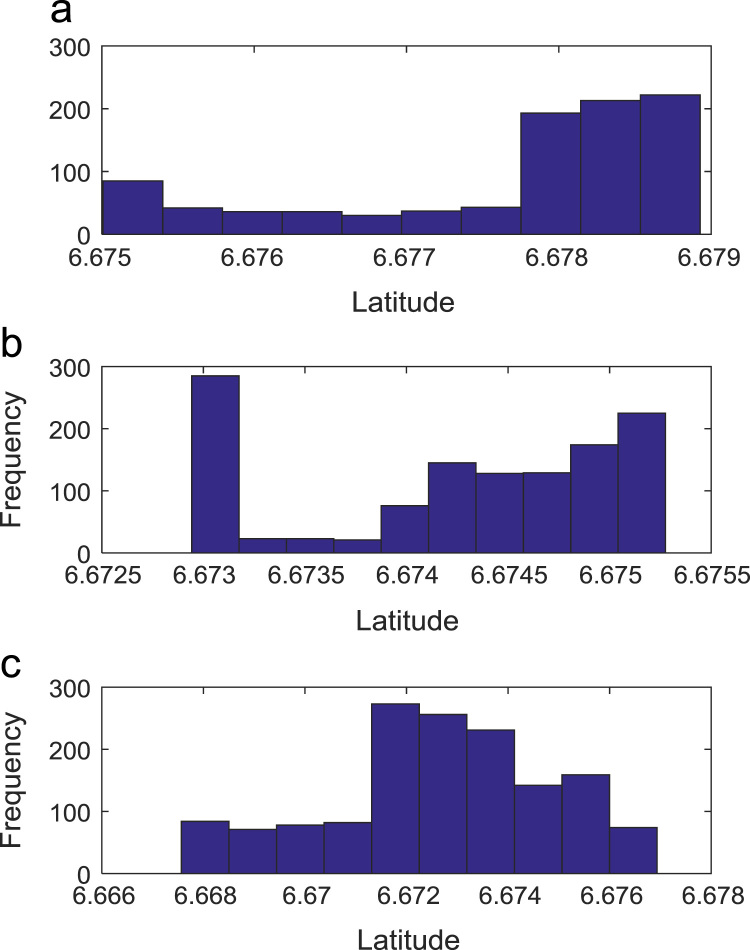
Frequency distribution of latitude data along Survey Route (a) X (b) Y and (c) Z.

**Fig. 3:**
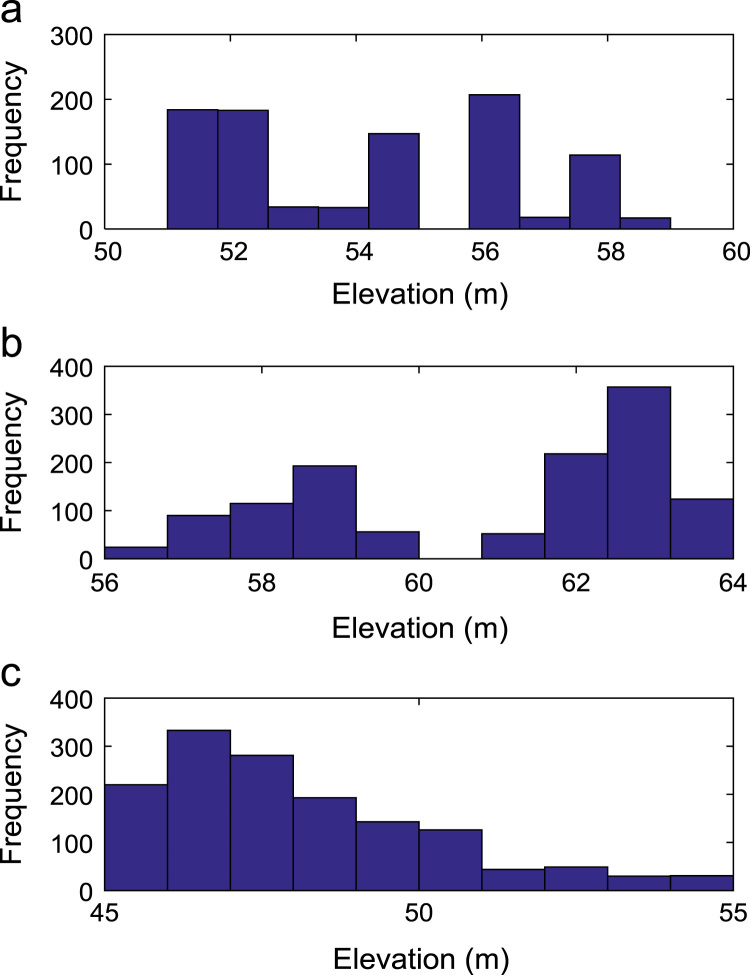
Frequency distribution of elevation data along Survey Route (a) X (b) Y and (c) Z.

**Fig. 4:**
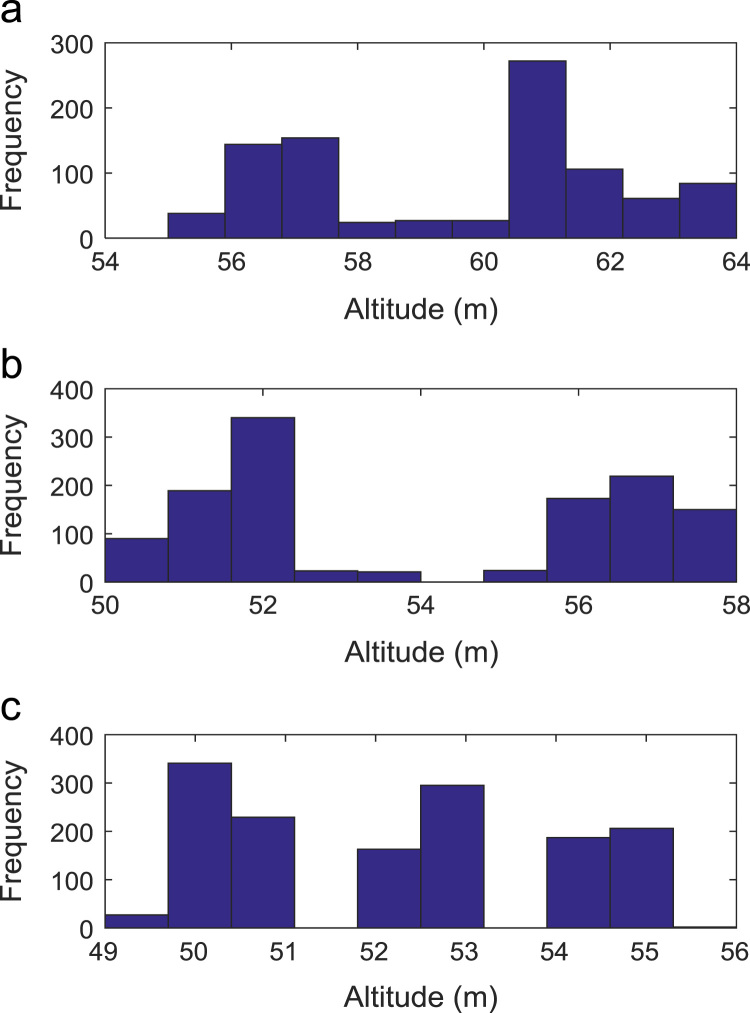
Frequency distribution of altitude data along Survey Route (a) X (b) Y and (c) Z.

**Fig. 5:**
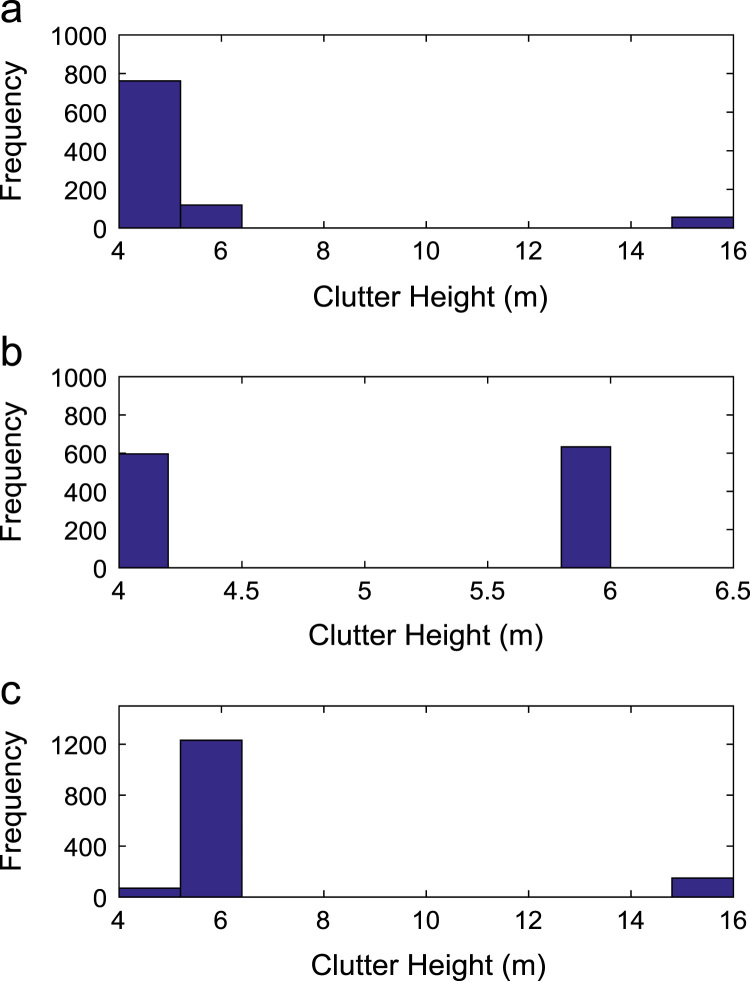
Frequency distribution of clutter height data along Survey Route (a) X (b) Y and (c) Z.

**Fig. 6:**
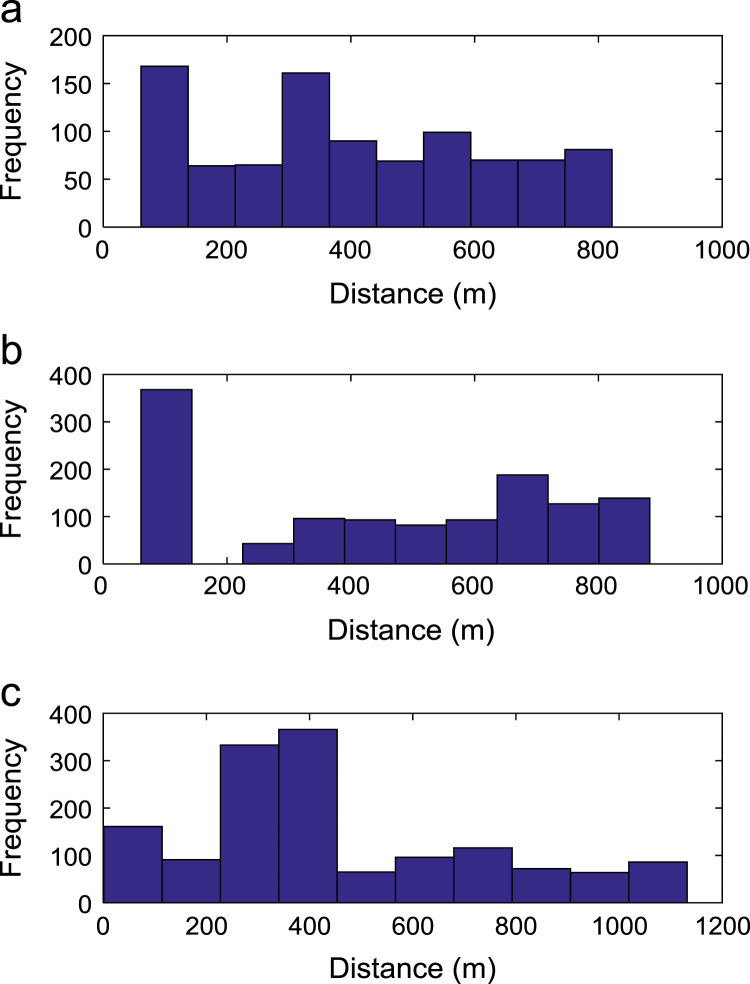
Frequency distribution of distance data along Survey Route (a) X (b) Y and (c) Z.

**Fig. 7:**
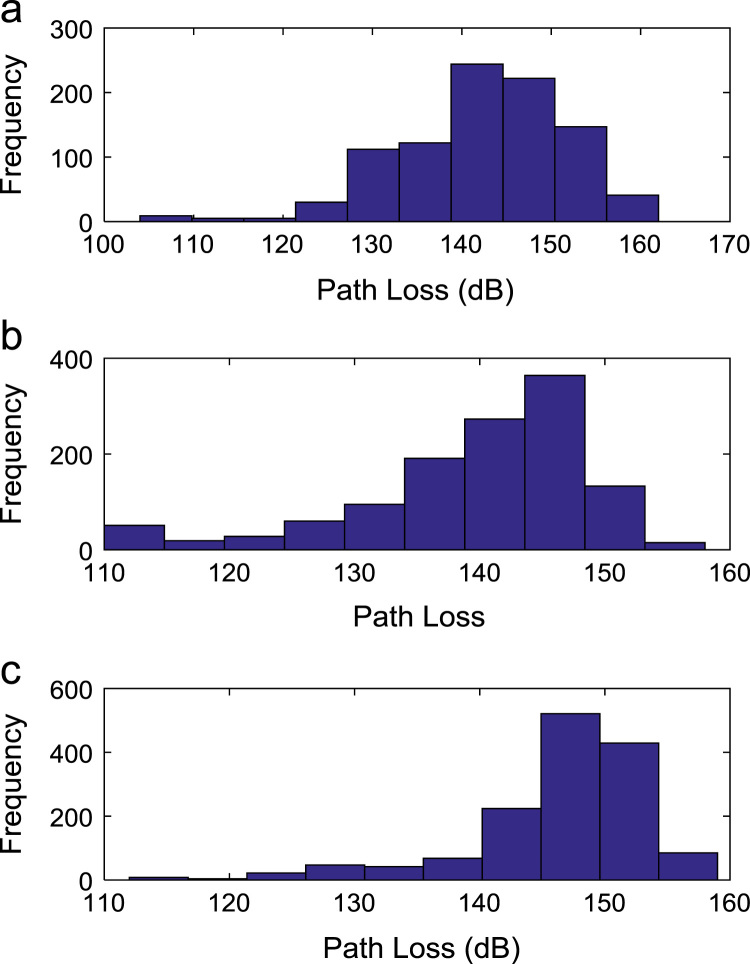
Frequency distribution of path loss data along Survey Route (a) X (b) Y and (c) Z.

Path loss may be defined as the difference in the transmitted signal power and the received signal power at varying separation distances between the transmitting antenna and the receiving antenna. Measurement campaigns were conducted along three survey routes within Covenant University, Ota, Ogun State, Nigeria. The path loss data and the terrain information about the smart campus environment are carefully explored in this data article. The terrain profile information available in this data article include: longitude; latitude; elevation; altitude; clutter height; and distance of separation between the transmitter and the receiver. These useful information are extracted from the Digital Terrain Map (DTM) of the study area. Detailed exploration of the dataset will facilitate the development of empirical models for radio wave propagation in smart campus environment. The descriptive first-order statistics of data obtained along Survey Route X, Y, and Z are presented in Table 1, Table 2, Table 3 respectively. For each of the routes under investigation, the results obtained showed that the statistics of the path losses differ as well as those of terrain profile data. Also, Fig. 1, Fig. 2, Fig. 3, Fig. 4, Fig. 5, Fig. 6, Fig. 7 show the frequency distributions of longitude, latitude, elevation, altitude, clutter height, distance, and path loss along the three routes.

**Table 1:** Descriptive first-order statistics of data obtained along Survey Route X.

	**Longitude**	**Latitude**	**Elevation (m)**	**Altitude (m)**	**Clutter height (m)**	**Distance (m)**	**Path loss (dB)**
Mean	3.1651	6.6777	54.22	59.68	4.97	399.81	142.42
Median	3.1644	6.6781	55.00	61.00	4.00	374.00	144.00
Mode	3.1635	6.6750	56.00	61.00	4.00	62.00	144.00
Standard Deviation	0.0021	0.0012	2.48	2.78	2.86	228.31	9.42
Variance	0.0000	0.0000	6.16	7.75	8.18	52,125.34	88.83
Kurtosis	1.8467	2.7316	1.68	1.66	13.26	1.91	4.44
Skewness	0.5506	− 1.0334	0.10	− 0.16	3.41	0.14	− 0.79
Range	0.0064	0.0039	8.00	9.00	12.00	761.00	58.00
Minimum	3.1628	6.6750	51.00	55.00	4.00	61.00	104.00
Maximum	3.1692	6.6789	59.00	64.00	16.00	822.00	162.00
Sample size	937	937	937	937	937	937	937

**Table 2:** Descriptive first-order statistics of data obtained along Survey Route Y.

	**Longitude**	**Latitude**	**Elevation (m)**	**Altitude (m)**	**Clutter height (m)**	**Distance (m)**	**Path loss (dB)**
Mean	3.1669	6.6742	61.03	54.00	5.03	460.49	139.72
Median	3.1672	6.6744	62.00	52.00	6.00	488.00	141.00
Mode	3.1635	6.6750	63.00	52.00	6.00	138.00	141.00
Standard Deviation	0.0024	0.0008	2.33	2.80	1.00	272.72	9.52
Variance	0.0000	0.0000	5.43	7.83	1.00	74,376.75	90.55
Kurtosis	1.5297	1.7995	1.86	1.37	1.00	1.56	4.50
Skewness	− 0.1321	− 0.4817	− 0.49	0.13	− 0.06	− 0.10	− 1.25
Range	0.0071	0.0023	8.00	8.00	2.00	822.00	48.00
Minimum	3.1634	6.6729	56.00	50.00	4.00	61.00	110.00
Maximum	3.1706	6.6753	64.00	58.00	6.00	883.00	158.00
Sample size	1229	1229	1229	1229	1229	1229	1229

**Table 3:** Descriptive first-order statistics of data obtained along Survey Route Z.

	**Longitude**	**Latitude**	**Elevation (m)**	**Altitude (m)**	**Clutter height (m)**	**Distance (m)**	**Path loss (dB)**
Mean	3.1600	6.6727	48.61	52.21	6.93	447.42	146.34
Median	3.1604	6.6728	48.00	52.00	6.00	356.00	147.50
Mode	3.1584	6.6720	47.00	50.00	6.00	356.00	147.00
Standard Deviation	0.0020	0.0022	2.23	1.80	3.10	288.35	7.30
Variance	0.0000	0.0000	4.98	3.24	9.61	83,144.89	53.29
Kurtosis	1.9779	2.7143	3.37	1.73	7.58	2.69	6.20
Skewness	− 0.3687	− 0.4146	0.92	0.10	2.51	0.76	− 1.53
Range	0.0069	0.0094	10.00	7.00	12.00	1131.00	47.00
Minimum	3.1559	6.6676	45.00	49.00	4.00	1.00	112.00
Maximum	3.1629	6.6769	55.00	56.00	16.00	1132.00	159.00
Sample size	1450	1450	1450	1450	1450	1450	1450

## 2 Experimental design, materials and methods

**Fig. 8:**
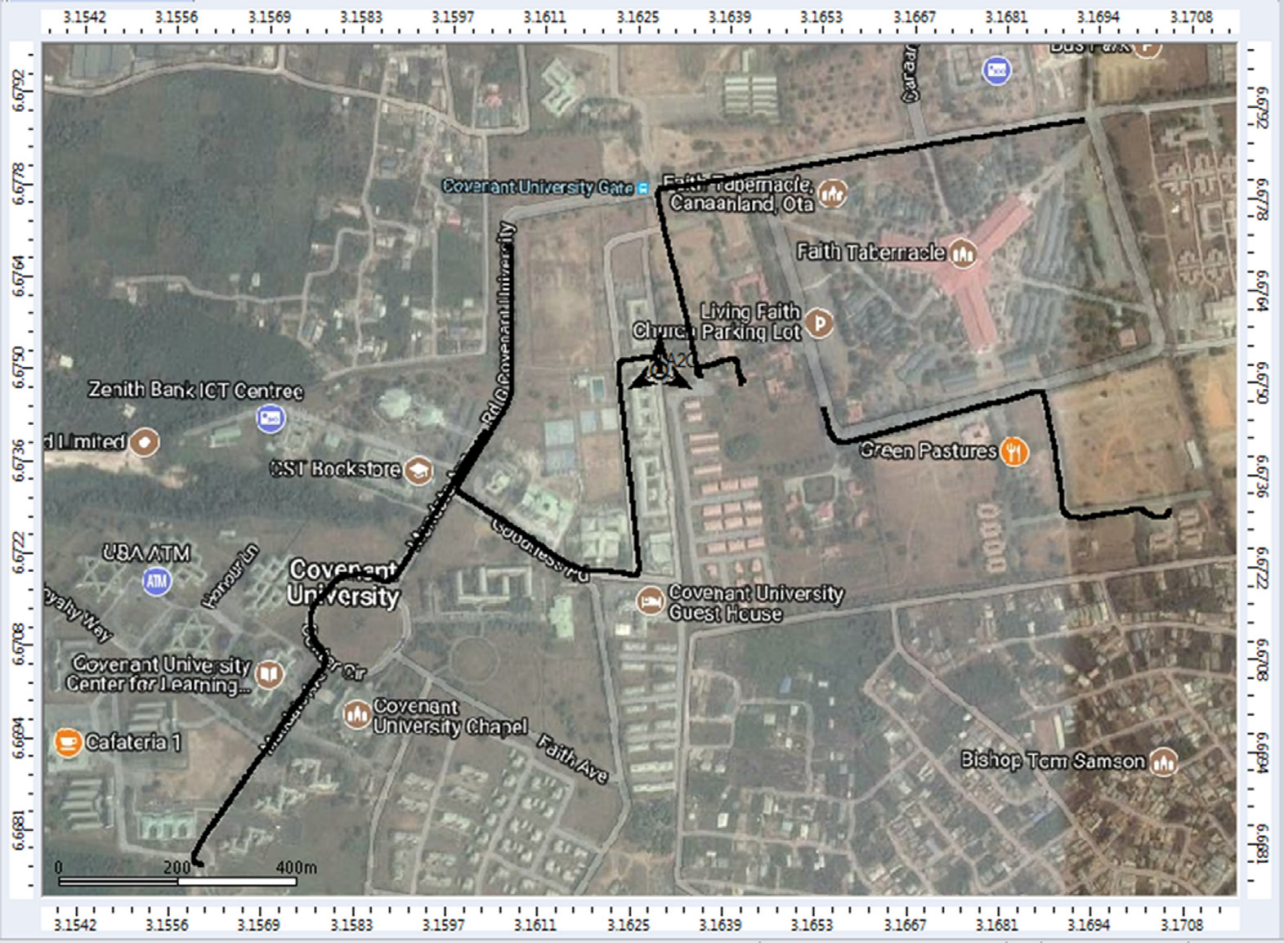
Digital Terrain Map (DTM) of the study area with measurement points.

**Fig. 9:**
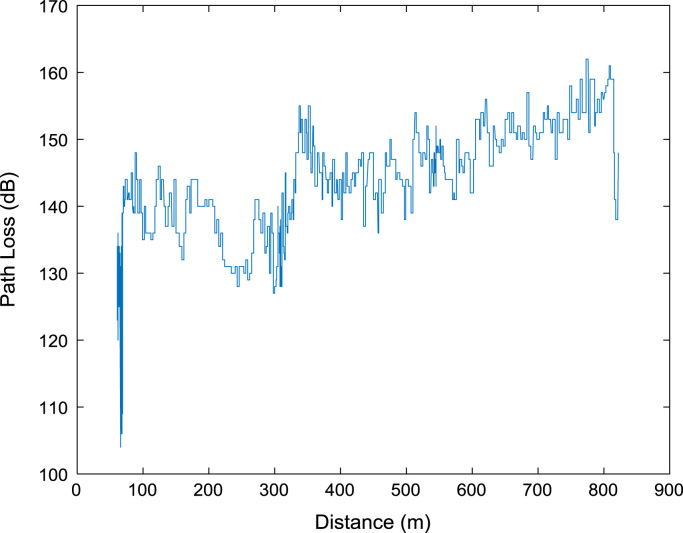
Plot of path loss against distance along Survey Route X.

**Fig. 10:**
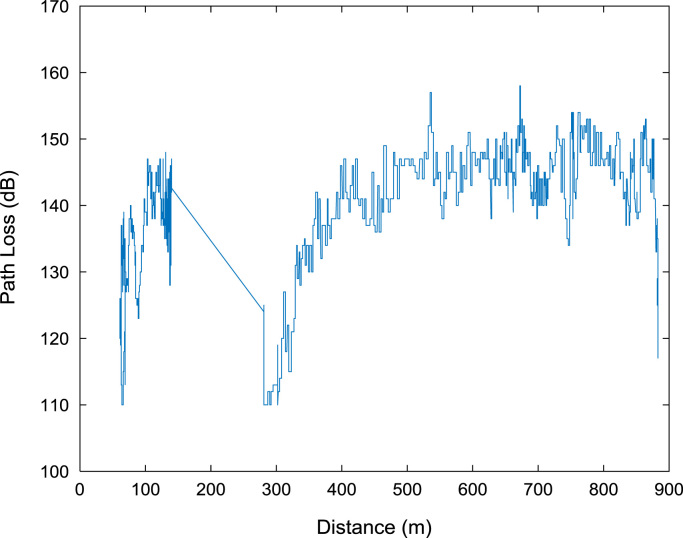
Plot of path loss against distance along Survey Route Y.

**Fig. 11:**
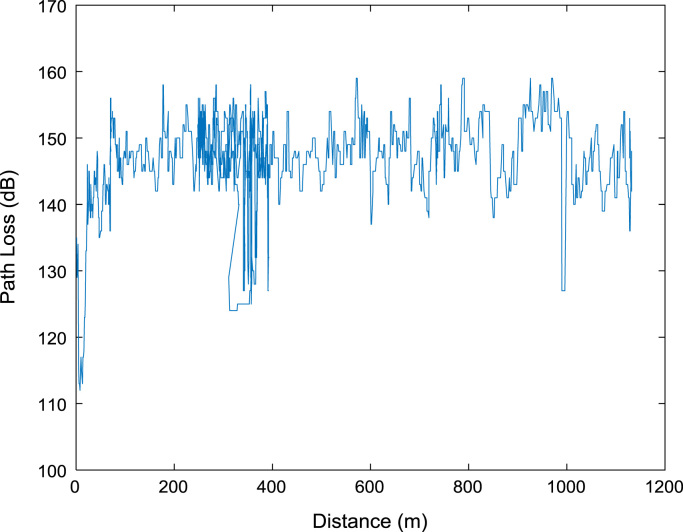
Plot of path loss against distance along Survey Route Z.

Extensive drive test measurements are performed along three different routes (X, Y, and Z) within Covenant University, Ota, Ogun State, Nigeria (Latitude 6°40′30.3″N, Longitude 3°09′46.3″E) to record path loss data as the mobile receiver moves away from each of the three 1800 MHz base station transmitters involved. The signal path loss data were collected with an experimental setup of a Test Mobile Station (TEMS) Sony Ericsson W995 handset, Ericsson TEMS Investigation software (version 9.0), Garmin Global Positioning System (GPS) receiver, and a Window-based Personal Computer (PC). The RF measurements were carried out under good climatic conditions. Also, good vehicular accessibility to site locations were considered for a smooth test drive. Distances covered by the drive routes were considered long enough to allow the noise floor of the receiver to be reached. The whole set-up was carefully placed in a vehicle, and the vehicle was driven at an average speed of 40 km/h. This speed was maintained to minimize Doppler effects. Also, the longitude, latitude, elevation, altitude, clutter height, and the distance information, which describes the smart campus environment, are obtained from Digital Terrain Map (DTM) in ATOLL radio network planning tool. The DTM of the study area is shown in Fig. 8. The map contains the measurement data points collected during the drive test. In Fig. 9, Fig. 10, Fig. 11, the values of the path loss data obtained were plotted against the corresponding distances. Correlation coefficients and their p-values for each of the seven network and terrain parameters are presented in matrix form in Table 4, Table 5, Table 6, Table 7, Table 8, Table 9. In this data article, correlation coefficient is said to be significant when an off-diagonal element of the *p*-Value matrix is smaller than the significance level of 0.05.

**Table 4:** Correlation Coefficient Matrix for Data on Survey Route X.

	Longitude	Latitude	Elevation	Altitude	Clutter height	Distance	Path loss
Longitude	1						
Latitude	0.7182	1					
Elevation	0.9004	0.8205	1				
Altitude	0.8639	0.8862	0.9603	1			
Clutter Height	0.2012	− 0.0730	0.1418	0.0761	1		
Distance	0.9381	0.9077	0.9157	0.9288	0.0946	1	
Path loss	0.7265	0.7142	0.7741	0.7549	0.0990	0.7581	1

**Table 5:** P-Value Matrix for Data on Survey Route X.

	Longitude	Latitude	Elevation	Altitude	Clutter height	Distance	Path loss
Longitude	1						
Latitude	0.0000	1					
Elevation	0.0000	0.0000	1				
Altitude	0.0000	0.0000	0.0000	1			
Clutter Height	0.0000	0.0254	0.0000	0.0198	1		
Distance	0.0000	0.0000	0.0000	0.0000	0.0037	1	
Path loss	0.0000	0.0000	0.0000	0.0000	0.0024	0.0000	1

**Table 6:** Correlation Coefficient Matrix for Data on Survey Route Y.

	Longitude	Latitude	Elevation	Altitude	Clutter Height	Distance	Path Loss
Longitude	1.0000	−0.8328	0.8226	0.8569	−0.4438	0.9994	0.5523
Latitude	−0.8328	1.0000	−0.5275	−0.6633	0.2671	−0.8511	−0.3032
Elevation	0.8226	−0.5275	1.0000	0.5571	−0.5188	0.8109	0.5937
Altitude	0.8569	−0.6633	0.5571	1.0000	−0.2209	0.8554	0.5565
Clutter Height	−0.4438	0.2671	−0.5188	−0.2209	1.0000	−0.4368	−0.0254
Distance	0.9994	−0.8511	0.8109	0.8554	−0.4368	1.0000	0.5434
Path loss	0.5523	−0.3032	0.5937	0.5565	−0.0254	0.5434	1.0000

**Table 7:** *P*-Value Matrix for Data on Survey Route Y.

	Longitude	Latitude	Elevation	Altitude	Clutter height	Distance	Path loss
Longitude	1						
Latitude	0.0000	1					
Elevation	0.0000	0.0000	1				
Altitude	0.0000	0.0000	0.0000	1			
Clutter Height	0.0000	0.0000	0.0000	0.0000	1		
Distance	0.0000	0.0000	0.0000	0.0000	0.0000	1	
Path loss	0.0000	0.0000	0.0000	0.0000	0.3736	0.0000	1

**Table 8:** Correlation Coefficient Matrix for Data on Survey Route Z.

	Longitude	Latitude	Elevation	Altitude	Clutter Height	Distance	Path Loss
Longitude	1						
Latitude	0.7512	1					
Elevation	0.2847	− 0.2099	1				
Altitude	0.0216	− 0.3494	0.5766	1			
Clutter Height	0.3521	0.3726	− 0.0236	0.3060	1		
Distance	− 0.9456	− 0.9012	− 0.0129	0.1929	− 0.3981	1	
Path loss	− 0.1663	− 0.0679	− 0.0894	− 0.1275	− 0.1376	0.1474	1

**Table 9:** *P*-Value Matrix for Data on Survey Route Z.

	Longitude	Latitude	Elevation	Altitude	Clutter height	Distance	Path loss
Longitude	1						
Latitude	0.0000	1					
Elevation	0.0000	0.0000	1				
Altitude	0.4108	0.0000	0.0000	1			
Clutter Height	0.0000	0.0000	0.3696	0.0000	1		
Distance	0.0000	0.0000	0.6243	0.0000	0.0000	1	
Path loss	0.0000	0.0097	0.0007	0.0000	0.0000	0.0000	1

## References

[1] Popoola S.I., Misra S., Atayero A.A. (2017). Outdoor path loss predictions based on extreme learning machine. Wirel. Personal. Commun..

[2] M.A. Salman, S.I. Popoola, N. Faruk, N. Surajudeen-Bakinde, A.A. Oloyede, L.A. Olawoyin, Adaptive Neuro-Fuzzy model for path loss prediction in the VHF band, in: Proceedings of the International Conference on Computing Networking and Informatics (ICCNI), 2017, pp. 1–6.

[3] Faruk N., Adediran Y.A., Ayeni A.A. (2013). Characterization of Propagation Path Loss at VHF and UHF bands for Ilorin City, Nigeria. Niger. J. Technol. (NIJOTECH) Univ. Nnsuka.

[4] N. Faruk, Y.A. Adediran, A.A. Ayeni, Error bounds of empirical path loss models at vhf/uhf bands in kwara state, Nigeria, in: Proceedings of the EUROCON, IEEE, 2013, pp. 602–607.

[5] Faruk N., Ayeni A., Adediran Y.A. (2013). On the study of empirical path loss models for accurate prediction of TV signal for secondary users. Prog. Electromagn. Res. B.

[6] Adeyemo Z.K., Ogunremi O.K., Ojedokun I.A. (2016). Optimization of okumura-hata model for long term evolution network deployment in Lagos, Nigeria. Int. J. Commun. Antenna Propag..

[7] Oseni O.F., Popoola S.I., Abolade R.O., Adegbola O.A. (2014). Comparative analysis of received signal strength prediction models for radio network planning of GSM 900 MHz in Ilorin, Nigeria. Int. J. Innov. Technol. Explor. Eng..

[8] Oseni O.F., Popoola S.I., Enumah H., Gordian A. (2014). Radio frequency optimization of mobile networks in Abeokuta, Nigeria for improved quality of service. Int. J. Res. Eng. Technol..

[9] Popoola S.I., Oseni O.F. (2014). Performance evaluation of radio propagation models on GSM network in urban area of Lagos, Nigeria. Int. J. Sci. Eng. Res..

[10] Popoola S.I., Oseni O.F. (2014). Empirical path loss models for GSM network deployment in Makurdi, Nigeria. Int. Ref. J. Eng. Sci..

[11] V.O. Matthews, Q. Osuoyah, S.I. Popoola, E. Adetiba, A.A. Atayero, C-BRIG: a network architecture for real-time information exchange in smart and connected campuses, in: Lecture Notes in Engineering and Computer Science: Proceedings of The World Congress on Engineering 2017, London, U.K., vol. 398–401, 5–7 July, 2017.

[12] S.I. Popoola, A.A. Atayero, N. Faruk, C.T. Calafate, E. Adetiba, V.O. Matthews, Calibrating the standard path loss model for urban environments using field measurements and geospatial data, in: Lecture Notes in Engineering and Computer Science: Proceedings of The World Congress on Engineering 2017, London, U.K., 5–7 July, 2017, pp. 513–518.

[13] S.I. Popoola, A.A. Atayero, N. Faruk, C.T. Calafate, L.A. Olawoyin, V.O. Matthews, Standard propagation model tuning for path loss predictions in built-up environments, in: Proceedings of the International Conference on Computational Science and Its Applications, 2017, pp. 363–375.

[14] S.I. Popoola, J.A. Badejo, S.O. Ojewande, A. Atayero, Statistical evaluation of quality of service offered by GSM network operators in Nigeria, in: Lecture Notes in Engineering and Computer Science: Proceedings of The World Congress on Engineering and Computer Science 2017, San Francisco, USA, 25–27 October, 2017, pp. 69–73.

[15] Popoola S.I., Atayero A.A., Faruk N., Badejo J.A. (2018). Data on the key performance indicators for quality of service of GSM networks in Nigeria. Data Brief.

[16] Popoola S.I., Atayero A.A., Badejo J.A., John T.M., Odukoya J.A., Omole D.O. (2018). Learning analytics for smart campus: data on academic performances of engineering undergraduates in a Nigerian Private University. Data Brief.

[17] Popoola S.I., Atayero A.A., Okanlawon T.T., Omopariola B.I., Takpor O.A. (2018). Smart campus: data on energy consumption in an ICT-Driven University. Data Brief.

[18] Popoola S.I., Atayero A.A., Faruk N. (2018). Received signal strength and local terrain profile data for radio network planning and optimization at GSM frequency bands. Data Brief.

[19] I.Y. Abdulrasheed, N. Faruk, N.T. Surajudeen-Bakinde, L.A. Olawoyin, A.A. Oloyede, S.I. Popoola, Kriging Based Model for Path Loss Prediction in the VHF Band, in: Proceedings of the 3rd International Conference on ElectroTechnology for National Development, Federal University of Technology, Owerri (FUTO), Imo State, Nigeria, 7–10 November, 2017, pp. 173–176.

[20] I.A. Sikiru, N. Faruk, S.I. Popoola, Y. Imam-Fulani, A.A. Oloyede, L.A. Olawoyin, et al., Effects of detection threshold and frame size on duty cycle in GSM bands, in: Proceedings of the 3rd International Conference on Electro-Technology for National Development, Federal University of Technology, Owerri (FUTO), Imo State, Nigeria, 7–10 November, 2017, pp. 343–346.

